# Using DNA metabarcoding to investigate honey bee foraging reveals limited flower use despite high floral availability

**DOI:** 10.1038/srep42838

**Published:** 2017-02-16

**Authors:** Natasha de Vere, Laura E. Jones, Tegan Gilmore, Jake Moscrop, Abigail Lowe, Dan Smith, Matthew J. Hegarty, Simon Creer, Col R. Ford

**Affiliations:** 1 National Botanic Garden of Wales, Llanarthne, Carmarthenshire, SA32 8HG, UK; 2 Institute of Biological, Environmental & Rural Sciences (IBERS), Aberystwyth University, Penglais, Aberystwyth, Ceredigion, SY23 3DA, UK; 3 Molecular Ecology and Fisheries Genetics Laboratory, School of Biological Sciences, Environment Centre Wales, Bangor University, Gwynedd, LL57 2UW, UK

## Abstract

Understanding which flowers honey bees (*Apis mellifera*) use for forage can help us to provide suitable plants for healthy honey bee colonies. Accordingly, honey DNA metabarcoding provides a valuable tool for investigating pollen and nectar collection. We investigated early season (April and May) floral choice by honey bees provided with a very high diversity of flowering plants within the National Botanic Garden of Wales. There was a close correspondence between the phenology of flowering and the detection of plants within the honey. Within the study area there were 437 genera of plants in flower during April and May, but only 11% of these were used. Thirty-nine plant taxa were recorded from three hives but only ten at greater than 1%. All three colonies used the same core set of native or near-native plants, typically found in hedgerows and woodlands. The major plants were supplemented with a range of horticultural species, with more variation in plant choice between the honey bee colonies. We conclude that during the spring, honey bees need access to native hedgerows and woodlands to provide major plants for foraging. Gardens provide supplementary flowers that may increase the nutritional diversity of the honey bee diet.

Pollination is a key regulating ecosystem service^1^,^2^ with 78% of flowering plants in temperate areas relying on animal pollination^3^. Globally, 75% of crops depend to some extent on animal pollination^4^, with vegetables and fruits being the most dependent on insects^5^.

Honey bees contribute both directly and indirectly to humans; through honey and wax, and as the pollinator of both wild and crop plants. The ease with which honey bees can be managed, compared with other pollinators, makes them crucial to crop pollination^6^. Honey bees increase yield in 96% of animal-pollinated crops^4^,^7^.

There is therefore considerable concern worldwide over increased rates of honey bee colony loss due to poor health^8^,^9^. Colony loss is caused by a number of interacting biotic and abiotic factors, exacerbated by apicultural mismanagement^10^. Pests and diseases, a reduction in suitable habitat for foraging, decreasing genetic diversity and exposure to agrochemicals all place mounting pressure on honey bee colonies. Intensive farming leads to reductions in flower-rich habitat and increases in mass-flowering crops that provide a single floral resource over a limited period of time^7^,^10^,^11^. Nutritional stress resulting from a lack of suitable foraging habitat may also combine with other factors to cause ill health and colony loss in honey bees^10^,^12^,^13^.

Recommendations for improving honey bee health include: reducing exposure to insecticides; preventing and limiting the spread of disease and providing a greater diversity of floral resources throughout the year^10^. On managed agricultural land, suggestions include maintaining areas of semi-natural habitat, sowing flower-rich field margins and creating and maintaining hedgerows^10^,^11^.

Gardens and other amenity areas can also provide valuable foraging resources for honey bees^14^. Horticultural plantings can provide a high diversity of floral resources and there are a number of schemes that provide lists of ‘pollinator friendly’ plants^15^,^16^,^17^. However, whilst some of these lists are based on careful observation over many years, others are based on anecdotal information and generally lack a firm evidence base^17^. In order to provide appropriate floral resources, a greater understanding is required of the foraging preferences of honey bees and how this relates to their nutritional needs^18^.

Honey bee nutritional requirements are met by nectar and pollen^19^. Nectar provides sugars that are processed into honey for longer-term storage, thereby providing the major source of energy for honey bees. Nectar from different plants varies in sugar type, concentration and also contains a range of other trace elements. Pollen provides honey bees with protein, which is of particular importance during brood rearing. Pollen varies greatly in its protein content and amino acid composition; it also contains varying amounts of lipids, carbohydrates, vitamins and minerals that are an important part of the honey bee diet^20^. Pollen diversity and quantity is also known to affect disease tolerance and longevity in honey bees^21^,^22^,^23^,^24^.

Early foraging success during spring is critically important during the honey bee life-cycle. Over winter, brood production starts and honey bees are reliant on honey and pollen stores from the previous year to provide sufficient energy resources for winter activities^19^. As soon as seasonal temperatures become warm enough and spring flowers begin to bloom, honey bees start to forage for nectar and pollen in order to replenish their stores and continue brood-rearing^19^. Frequent low spring temperatures and rain prevent foraging and subsequently lead to a reduction or complete cessation in brood production, jeopardizing the survival of the colony^25^. Particularly in northern temperate climates, early spring remains the period during which pollen shortages occur most frequently^25^.

Honey bees are often described as ‘super-generalists’ utilizing a wide range of flowering plants^7^,^26^. However, Hawkins *et al*.^27^ found that although honey bees use a wide taxonomic range, actual resource use was represented by a comparatively small number of core species. Knowing which plants honey bees forage upon is critical to understanding the link between floral availability, honey bee nutrition and health^28^.

Honey bee activity has been studied using a range of methods including interpretation of bee dances^29^, mark recapture^30^,^31^,^32^, and harmonic radar^33^,^34^. These methods can be used to track where honey bees go but do not provide an assessment of which plants are visited.

Methods used to assess floral visitation include identifying the pollen returned to the hive by honey bees, either directly by collecting pollen at the hive entrance using ‘pollen traps’ or through examining pollen found within honey^35^,^36^. Identifying pollen returned to the hive provides a direct measure of pollen foraging, whilst the pollen within honey provides a longer-term overview of the plants used for both nectar and pollen^27^,^36^. Such pollen assessments have typically been used to identify the botanical composition of honey in order to check its geographic origin for food quality and traceability purposes and have more rarely been used to investigate foraging preferences^37^.

The traditional method for identification of honey bee collected pollen uses light microscopy to characterize morphological features and requires considerable skill and experience. Furthermore, pollen from a number of plant species can be particularly difficult to discriminate^27^,^38^,^39^. An alternative is to use DNA-based identification, treating the honey-derived pollen as a source of environmental DNA^40^,^41^,^42^. This can increase the level of species discrimination, reduce processing time and does not require the high level of taxonomic expertise required for microscopic examination^27^,^42^,^43^,^44^. A DNA metabarcoding approach, combining the amplification of universal markers with high throughput sequencing, importantly provides the opportunity to analyse many samples, containing mixtures of species, with extensive depth of sequencing coverage and associated ecological insights^45^,^46^. Hawkins *et al*.^27^ recently demonstrated the utility of using DNA metabarcoding of honey in order to assess honey bee foraging preferences. The *rbcL* DNA barcode marker was used as this provides a very high level of universality of taxon amplification, ensuring that the greatest number of species within the sample are amplified and sequenced^27^,^47^. The ability to identify unknown sequences is dependent on the quality and comprehensiveness of the reference library. Within the UK, native flowering plants have been DNA barcoded using the *rbcL* DNA barcode marker by de Vere *et al*.^48^.

Here, we investigate honey bees’ actual plant resource use if they are provided with a very high diversity of flowering plants. The National Botanic Garden of Wales, UK, contains over 8000 taxa of flowering plants, all of which are catalogued using a botanical garden collection management system (IRIS BG) so that their identity and location is recorded. We collected honey from three hives within the botanic garden’s apiary, at two successive dates during April and May (2015) and subjected the DNA obtained from the pollen within the honey to Illumina MiSeq metabarcoding sequencing of the *rbcL* DNA barcode marker. During the same period as honey collection, the botanic garden was divided into a number of survey zones and the presence of plant species in flower within these were recorded in order to provide a means of comparing flower choice with availability. Consequently, we present an approach that uses DNA metabarcoding of honey samples to provide a comparative characterisation of early-season honey bee foraging and resource use in the face of extensive floristic diversity.

## Results

**Figure 1:**
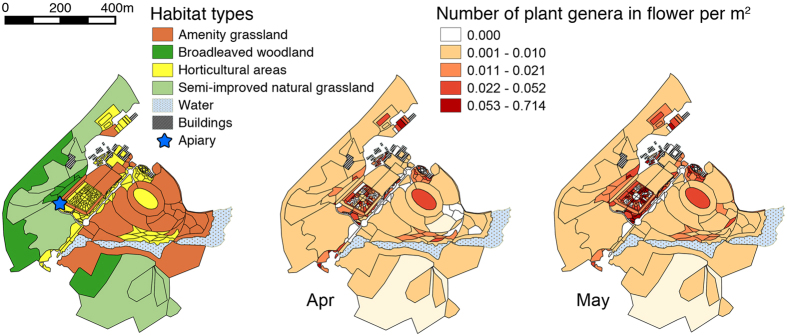
Survey area illustrating habitat types and the number of plant genera in flower per m^2^ for April and May. Maps created in QGIS v2.8.4. www.qgis.org.

The surveyed area was 34.2 ha and contained a range of native habitats, including semi-improved grassland (39%), woodland and hedgerows (15%) along with planted horticultural areas (13%) and amenity grassland (33%) (Fig. 1). The number of plant genera in flower per m^2^ tended to be higher in the horticultural areas compared to the native habitats (Fig. 1).

The apiary was located centre-left within the study site, close to semi-improved grassland, woodland and a richly-planted horticultural area which contained a systematics garden with flowering plants arranged according to the APG3 classification system^49^ (Fig. 1). The study site was within an agricultural landscape, which contained an organic farm and improved agricultural land used for grazing sheep and cattle.

Three hives within the apiary were used throughout the current study; hive A was the largest comprising 14 frames of bees in April and 21 in May. The smallest was hive B with eight frames of bees in April and 16 frames in May (Table 1).

Following the DNA metabarcoding procedure, Illumina MiSeq sequences were trimmed and merged, with only full-length reads over 450 bp used in downstream analysis. Sequences were compared against the Barcode UK database^48^ and GenBank using MegaBLAST, with 99.6% of reads identifiable to family, genus or species level (Supplementary Data SI 1).

**Figure 2:**
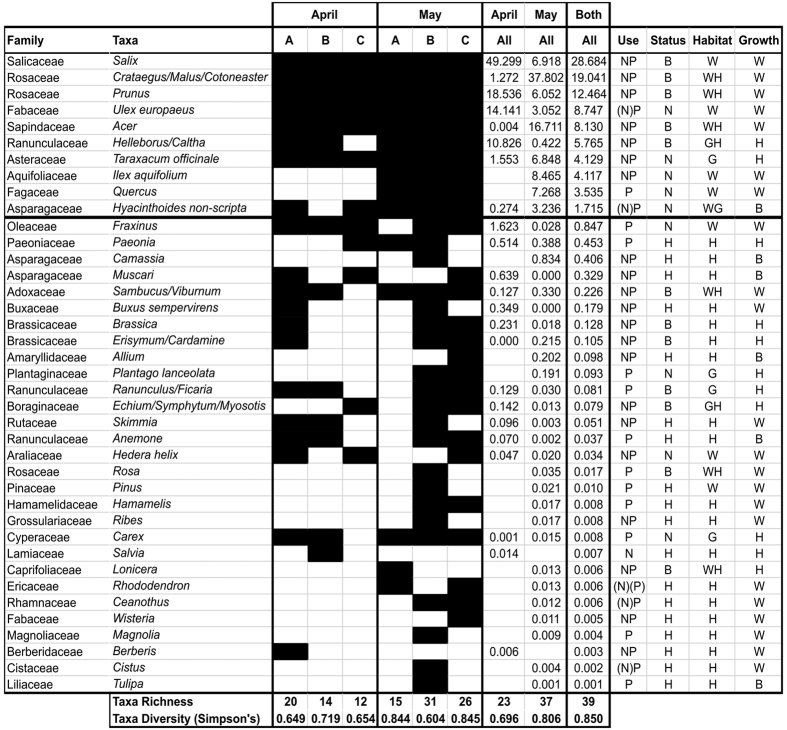
Plants found within honey collected in April and May from three honey bee colonies, along with the status and habitat of the recorded plants. (**A–C**) denote the three hives. For April and May the presence of plants within the honey sample is shaded in black for each hive. The proportion of DNA reads (%) for each plant is then given for all of the hives together, for April, May and both months combined. Supplementary Data SI 1 provides the proportions per hive. Use: whether honey bees use the plant for nectar N or pollen P according to Howes^50^, brackets denote that this plant is considered to be used infrequently for this purpose. Status of plant: native N, horticulture H, or both B. Plants that are designated as ‘both’ include native plants along with their horticultural relatives and varieties. These plants can be described as native and near-native. Habitat of the plant within the survey area: woodland, hedgerows and scrub W, grassland G, horticulture planting H. Growth form: woody tree or shrub W, herbaceous H, bulb or corm B. Taxa richness and diversity (Simpson’s Diversity Index) are provided for each colony separately and combined for each month.

In total, 39 taxa were identified across the three hives in April and May (Fig. 2). Most (67%) were identified to genus level, 18% were identified to species and 15% to family. The family level designations were identified to two or three closely related genera, for example *Malus, Cotoneaster* and *Crataegus*.

In April, 23 taxa were identified across all three hives, this increased to 37 taxa in May (Fig. 2). The hives varied in the number of taxa recorded ranging from 12 to 20 in April and 15 to 31 in May. There was no significant difference in taxon richness between the months (Mann-Whitney U, Z = −1.528, p = 0.127, N = 3). The diversity of taxa over all of the hives (assessed using Simpson’s Diversity Index) was higher in May (0.806) compared to April (0.696) (Fig. 2). This was variable between the hives, ranging from 0.649 to 0.719 in April and 0.604 to 0.845 in May. The difference in Simpson’s Diversity between the months was not significant (Mann-Whitney U, Z = −0.655, p = 0.513, N = 3).

Examining the results for April and May together we find that 96% of the DNA reads were represented by just ten taxa, whilst the remaining 29 taxa comprised the remaining 4% of reads. In April, only seven out of 23 (30%) of taxa occurred at greater than 1% and in May, nine out of 37 (19%) occurred at more than 1%. The major plants in April were *Salix* spp., followed by *Prunus* spp., *Ulex europaeus, Helleborus/Caltha, Fraxinus* spp.*, Taraxacum officinale* and the *Crataegus/Malus/Cotoneaster* group. In May, the major plants were *Crataegus/Malus/Cotoneaster*, followed by *Acer* spp., *Ilex aquifolium, Quercus* spp., *Salix* spp., *Taraxacum officinale, Prunus* spp., *Hyacinthoides non-scripta* and *Ulex europaeus*.

Each of the three honey bee colonies makes an independent decision on which plants to forage upon^19^ and so it is interesting to note that in April there were significant correlations in the abundance of plants used by the honey bees from hive A compared to B (Spearman’s Rho = 0.649, p-value = 0.0001, N = 39) and A compared to C (Spearman’s Rho = 0.528, p-value 0.001, N = 39). In May, there were significant correlations between plant abundance between all three hives (Spearman’s Rho A & B = 0.660, A & C = 0.670 and B & C = 0.537, all p-values 0.0001, N = 39). These remain significant even when allowing for Bonferroni correction for multiple testing (Supplementary Data SI 1).

The major taxa tended to be used by all three hives, whilst the taxa occurring at lower levels tended to be used by one or two hives. In April, the seven taxa occurring at greater than 1% were used by all three hives, with the exception of *Helleborus/Caltha* which were only used by two hives. Out of the taxa occurring at less than 1% only 6% were used by all three hives. In May, all nine taxa with abundance greater than 1% were used by all three hives, whilst only 11% of those with abundance less than 1% were used by all three hives (Fig. 2).

The plants recorded within the honey are mostly used for nectar and pollen, although some are used for pollen only^50^ (Fig. 2). The major plants used were often woodland or hedgerow plants (40%) whilst the minor plants tended to be horticultural taxa (66%)(Fig. 2 and Supplementary Data SI 1). Major plants included the trees and shrubs, *Salix, Crataegus, Malus, Prunus, Acer, Ulex europaeus, Ilex* and *Quercus*, along with herbs *Taraxacum officinale* and *Hyacinthoides non-scripta*. Minor plants included horticultural taxa such as the spring-flowering trees and shrubs *Lonicera, Rhododendron, Wisteria, Ceanothus, Hamamelis, Viburnum, Magnolia, Skimmia, Ribes, Cistus, Berberi*s and *Paeonia*. Spring bulbs and corms were also well represented including: *Allium, Muscari, Camassia, Anemone* and *Tulipa*.

The major plants found at greater than 1% in the honey were all native (50%) or native and near-native (50%) plants, whilst a much greater proportion of the minor plants were horticultural species (62%) with only 14% native plants. This difference was statistically significant (Fisher’s Exact test, p-value = 0.001) (Fig. 2 and Supplementary Data SI 1).

All of the plants found within the honey samples were recorded as flowering within the study site, with the exception of four taxa. Three of these, *Carex, Pinus* and *Buxus sempervirens* were present within the study site and flower during the spring, and so their absence may reflect that their flowering was missed during the floral surveys. The fourth species *Hedera helix,* was found in many areas of the study site but flowers in autumn and winter rather than spring. It is likely that the presence of this species within the honey had been carried over from the end of last season.

Comparing plants used by the honey bees to the plants in flower within the survey area showed that the honey bees used only a very small proportion of the flowering plant taxa. There were 80 plant families in flower during April and 85 in May and the honey bees used 23% and 34% of these respectively. There were 291 plant genera flowering in April and 360 in May and of these, the honey bees used 11% and 13% respectively (Table 2 and Supplementary Data SI 2).

**Figure 3:**
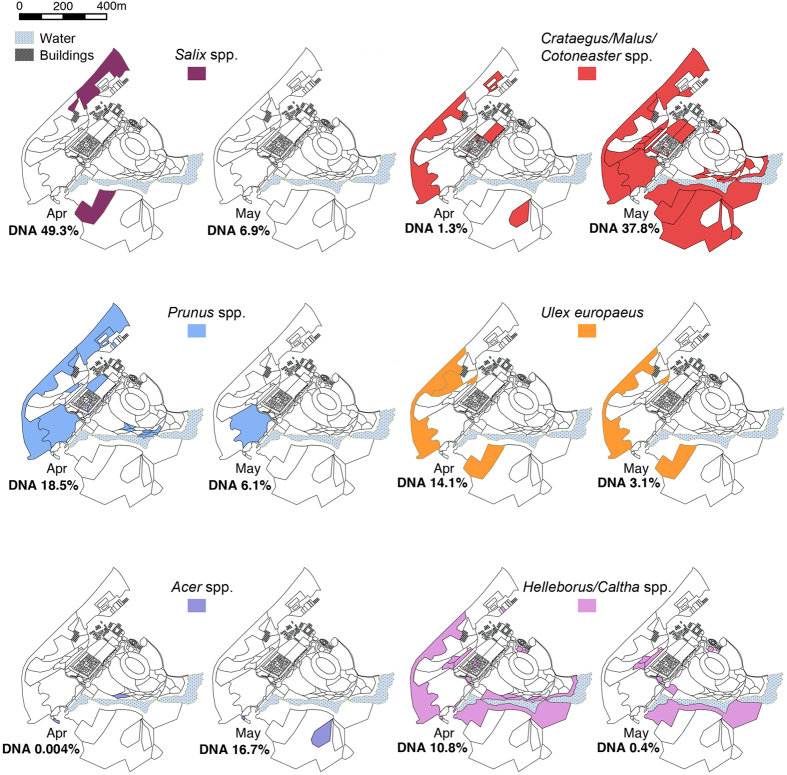
The location of plants in flower during April and May within the study site and proportion of that plant’s DNA (%) found within the honey samples using DNA metabarcoding. Maps are shown for all the plants with a DNA percentage of greater than 5% in the honey samples, with the results for all three hives combined. Maps created in QGIS v2.8.4. www.qgis.org.

The plants used by the honey bees and their abundance within the honey closely corresponded to the flowering phenology within the survey area (Fig. 3). *Salix, Prunus* and *Helleborus/Caltha* were predominantly recorded in the honey during April, corresponding with peak flowering times within the study site. The *Crataegus/Malus/Cotoneaster* group occurred at high abundance in the honey during May, when these plants were flowering prolifically in many areas of the site. *Acer* spp. were recorded at a low level in the honey during April but higher levels in May, corresponding to when the trees began to flower. *Ulex europaeus* was an exception as it was more frequent in the April honey even though it was flowering in both months.

The different survey areas within the study site that a plant was recorded as flowering within can be used to calculate an approximate area of occurrence that can be related to the abundance of plant DNA recorded for each hive (Supplementary Data SI 1). There were no significant correlations between the area of occurrence and DNA abundance for any of the three hives for April and May (Supplementary Data SI 1). Of the major plants used by the three hives some of these were widely distributed within the study site but not all of them. It should be highlighted however that it was only the distribution of the plants in the study site that was assessed and not their abundance.

## Discussion

Within this study, honey bees had close range access to a very high diversity of native and horticultural plants, but made use of only a very small proportion of the species in flower and an even smaller number were frequently recorded within the honey. According to our data, it appears that honey bees in the spring have a staple diet of a small number of core plant species that are supplemented with a range of horticultural plants used at lower levels.

The plants recorded within the honey closely follow the availability of the plants within the study site, which would indicate a foraging distance of less than 1 km. However, the honey bees may have travelled further away to forage on the same species of plants not mapped during this exercise. Honey bees have been recorded travelling over 10 km to forage^51^, but the energetic cost of extensive foraging distances means that they are more likely to use floral resources that are closer to the hive^52^,^53^.

The major plant taxa represented in the honey (over 1%) in April and May from all three hives were the woody trees and shrubs; *Salix, Crataegus/Malus/Cotoneaster, Prunus, Ulex europaeus, Acer, Ilex aquilinum* and *Quercus,* along with the herbs; *Helleborus/Caltha, Taraxacum officinale* and *Hyacinthoides non-scripta.* These are all either native or near-native plants to the UK and are mostly found in hedgerows and woodland. The minor plants were more frequently horticultural taxa including trees, shrubs, herbaceous plants, along with bulbs and corms.

Each colony of honey bees makes an independent choice amongst the resources available^19^. Here we show that all three hives choose the same major plants. There is also a high degree of similarity between the major taxa recorded within this study and other studies that have examined seasonal plant use of honey bees within the British Isles using microscopic examination of pollen. Coffey and Breen^54^ recorded *Salix, Ulex, Ranunculus, Prunus, Acer, Fagus sylvatica, Quercus, Ceanothus, Sorbus* and *Crataegus* during April and May in Ireland; we find eight of these 10 taxa in our results. McLellan^55^ found that honey bees in Scotland only used a mean of 14 plant species per colony with six types representing 85% of pollen collected. Trees and shrubs from mixed deciduous woodland were preferred with *Crataegus monogyna, Fagus sylvatica* and *Acer pseudoplatanus* being particularly important early in the season. Synge^35^ recorded major pollen early season sources in England as *Crataegus, Prunus, Acer, Berberis, Brassica, Quercus, Salix* and *Ulex europeaus*; we have also recorded all of these plants.

There are a number of reasons why certain plants may be particularly important for the honey bee diet. The abundance of plants within the landscape and their level and quality of nectar and pollen production are likely to be key factors. Baude *et al*.^11^ recorded that just 22 plants accounted for 90% of nectar production in native and agricultural habitats within the UK (horticultural habitats were not included). Of the 39 taxa we have recorded, nine were found on this list of 22, including the three plants found most abundantly within the honey: *Salix, Crataegus monogyna* and *Prunus*.

We did not find a significant relationship between the area of occurrence of plants within the study site and their DNA abundance within three hives. The area of occurrence used here was an approximate measure of plant distribution within the study site. A key area therefore for further work is to assess plant abundance along with distribution and relate this to which plants the honey bees use.

The plants used by the honey bees also reflect choices made at the individual flower level. Flower morphology affects which flowers honey bees can access, whilst nectar volume, concentration and sugar composition have all been shown to influence honey bee flower choice^28^. Some of the plants used by the honey bees have a high sugar concentration, such as *Acer* spp., *Hedera helix* and *Taraxacum officinale* but others such as *Hyacinthoides non-scripta* and *Ranunculus* spp. do not^11^.

Aronne *et al*.^37^ working on honey bees in Mediterranean ecosystems, suggested that foraging was driven more by pollen rather than nectar requirements. The plant taxa we have recorded within honey include species used for pollen or nectar, along with plants used for both resources. Anemophilous genera such as *Quercus* and *Fraxinus* that produce abundant pollen but no nectar feature within the honey at high frequency.

Maurizio^21^ showed that pollen from certain species helped honey bee development and longevity. *Plantago* spp. were beneficial for brood development whilst *Ranunculus* spp. pollen reduced longevity in caged bees. Pollen varies considerably in its protein content and amino acid composition and diets consisting of a single pollen source are generally considered insufficient for honey bee nutritional requirements^20^.

It has been suggested that honey bees select pollen higher in essential amino acids or obtain a balance of amino acids by collecting a diverse pollen diet^28^. Individual honey bees show preferences for collecting certain pollen types over others but these choices do not seem to be predicted by nutritional content^56^. Pernal and Currie^56^ showed however, that at the colony level, reductions in pollen quality or quantity within the hive resulted in an increase in recruitment of pollen foragers.

Pollen also provides the main lipid source for honey bees, with lipid levels within pollen ranging from 1 to 20%^28^. Entomophilous pollen has a lipid rich exterior (the pollenkitt) which is known to be an important component of honey bee diet and also acts as a discriminative stimulus between different pollen types^28^.

The pattern we have observed of a few major plants is consistent with honey bees using a small number of abundant, nectar and pollen-rich species to provide the bulk of carbohydrate, protein and lipid early in the season^19^,^57^. Our data suggest that these nutritional resources are supplemented with a range of minor, less intensively utilised species. These minor species may be required to provide additional essential nutrients lacking from the major sources of pollen and nectar. Further research is required to confirm this and also to investigate which combinations of plants are predicted to provide an optimal resource for honey bee health.

Including both the major and minor floral sources, the effective proportion of species utilised by the honey bees represents a very small percentage of the flowering plants that were available. The reason why only 11% of genera were used is a key area for further work. The honey sampled may not be an exhaustive list of all the plants that the honey bees were using. A 10 g sample of honey was removed from the comb and biases during DNA extraction and PCR mean that not all plants present at low levels may be detected^27^. It is therefore possible that some plants being foraged upon at low levels have been missed. Similarly, using the *rbcL* marker, the level of discrimination is most often at the generic level meaning that the results do not provide a complete list of forage species. Nevertheless, the very close correspondence between flowering phenology and detection within the honey shows that DNA metabarcoding provides a valuable tool for investigating honey bee foraging at a landscape scale.

The results have implications for managing habitats for honey bees. They suggest that honey bees have a taxonomically diverse but small number of core plant requirements that are supplemented with lower levels of other species. Current advice on honey bee foraging suggests providing a diverse floral resource^10^. Floral diversity is valuable in that it provides the range that honey bees may require, but in addition to this the quality and quantity of the resource is likely to be particularly important. In this study the honey bees were provided with a very high diversity of horticultural plants but their main foraging activity during the spring was not on these species. Instead it focused on native or near-native plants available within the study area, which were also very frequent within the wider agricultural landscape surrounding the study site. The foraging measured by metabarcoding honey corresponds closely both with other studies that have examined pollen use by honey bees and also the plants that provide the greatest abundance of nectar within the UK^11^. The major plants are characteristic of native woodlands and hedgerows, suggesting that whilst gardens provide an important source of additional plants, they cannot replace diverse semi-natural habitats.

## Methods

### Floral Surveys

The National Botanic Garden of Wales was divided into 20 survey zones and within each zone, individual flowerbeds were recorded and mapped using QGIS v2.14.3. (Supplementary Data SI 2). Floral surveys were carried out from April 27^th^ 2015 to May 1^st^ 2015 and again from May 25^th^ 2015 to May 29^th^ 2015. A list of plant species in flower was recorded within every flowerbed for each survey zone.

### Honey sampling and DNA extraction

Three colonies managed in the same way and with healthy queens were used. On April 30^th^ 2015 and May 27^th^ 2015, 30 ml of freshly deposited honey was collected from a comb within each hive using a sterile 50 ml tube to crush a small section of comb and release the honey. The wax was removed using sterile forceps and 10 g of honey weighed out for DNA extraction.

DNA was extracted from the honey using a modified version of the DNeasy Plant Mini extraction kit (Qiagen). The 10 g of honey was made up to 30 ml with sterile water and incubated in a water bath at 65 °C for 30 minutes. Samples were then centrifuged (Sorvall RC-5B) for 30 minutes at 15,000 rpm at room temperature.

The supernatant was discarded and the pellet resuspended in 400 μL AP1 from the DNeasy plant mini kit (Qiagen) and 80 μL proteinase K (1 mg/ml) (Sigma). This was incubated for 60 minutes at 65 °C in a water bath and then disrupted using a TissueLyser II (Qiagen) for 4 minutes at 30 Hz with 3 mm tungsten carbide beads. The remaining steps were carried out according to the manufacturer’s protocol, excluding the use of the QIAshredder and the second wash stage. The extracted DNA was purified using the OneStep PCR Inhibitor Removal Kit (Zymo Research).

### PCR and Sequencing

The *rbcL* DNA barcode was amplified via a two-step PCR protocol. A primary tailed amplification of the *rbcL* region was followed by a second round of amplification that annealed sample specific Illumina Nextera indices so that samples could be separated via bioinformatic processing. Samples were first amplified using the universal primers rbcLaf and rbcLr506^48^ to which 5′ overhangs had been added complementary to the Nextera index primers.

(rbcLaf + adaptor: **TCGTCGGCAGCGTCAGATGTGTATAAGAGACAG**ATGTCACCACAAACAGAGACTAAAGC

rbcLr506 + adaptor: **GTCTCGTGGGCTCGGAGATGTGTATAAGAGACAG**AGGGGACGACCATACTTGTTCA). Initial PCR used a final volume of 20 μl: 2 μl template DNA plus 10 μl of 2x Phusion Mastermix (New England Biolabs), 0.4 μl forward primer (rbcLa-F), 0.4 μl reverse primer (rbcLr506), and 7.2 μl of molecular biology grade water (Sigma). Thermal cycling conditions were: 95 °C for 2 minutes; 95 °C for 30 seconds, 50 °C for 1 minute 30 seconds, 72 °C for 40 seconds (35 cycles); 72 °C for 5 minutes, 30 °C for 10 seconds.

Products from the first PCR were purified following Ilumina’s 16 S Metagenomic Sequencing Library Preparation protocol^58^ using Agencourt AMPure XP beads (Beckman Coulter). The Index PCR stage used a 25 μl reaction (12.5 μl of 2x Phusion Mastermix, 2.5 μl of Nextera XT i7 Index Primer, 2.5 μl of Nextera XT i5 Index Primer, 5 μl of PCR grade water, and 2.5 μl of purified first-round PCR product). Following the Index PCR, a 1% gel was run to verify its success. The PCR clean-up 2 of the Illumina protocol was then followed, cleaning 20 μl of Indexed PCR product, with a 1:0.8 ratio of product to AMPure XP beads.

Amplified products were quantified using a Qubit fluorescence spectrophotometer (Life Technologies) and pooled at equal concentrations to produce the final library. This was again quantified via Qubit to determine concentration and adjusted to 10 nM concentration with 0.1 M Tris-HCl/0.01% Tween 20 solution prior to sequencing on an Illumina MiSeq platform. Library denaturation and sample loading steps followed the Illumina protocol: sample was loaded at 3 pM concentration with 20% PhiX control spike and paired-end sequences generated in 2 × 300 bp format.

### Data analysis

A data analysis pipeline was created to process the Illumina sequence reads and to match them to known taxa within a local reference database. Files containing the sequence reads used in this study are available through the NCBI sequence read archive (SRA accession **SRP069741**). The source code and tools used for the pipeline are available on github at https://github.com/colford/nbgw-plant-illumina-pipeline. Sequences were quality trimmed and then merged with only sequences greater than 450 bp used in downstream analysis. A local BLAST database was created from chloroplast sequence data from GenBank and *rbcL* sequences obtained from the Barcode Wales project, which provided 98% coverage for the native flowering plants of Wales^48^. DNA sequences from the honey were scored against the database using Megablast. If the sequence top bit score matched to a single species, then the sequence was identified to that species. If the top bit score was the same for different species belonging to the same genus, then the result was attributed to the genus level. If the top bit score belonged to multiple genera within the same family then a family level designation was made. Sequences blasting to multiple families were considered to be unknown^27^. Results for each honey sample were manually filtered so that only species recorded within the UK were retained. Stace^59^ and Cubey and Merrick^60^ were used as references for plants occurring in the UK as natives, aliens, in horticulture or agriculture.

The number of sequence reads for the plant taxa in each honey sample were converted into proportions (%) for downstream analysis. This was used as a measure of DNA abundance. DNA metabarcoding may not provide a robustly quantitative measure of DNA. Differences in the amount of pollen produced by different plants, possible variations in plastid copy number, along with biases during DNA extraction, PCR and sequencing may affect the results^27^,^44^. Hawkins *et al*.^27^ compared microscopy with 454-sequencing of honey and showed that the most abundant taxa were the same with both methods. Kraaijeveld *et al*.^44^ showed a good correlation between microscopy based pollen counts and DNA sequence reads for airborne pollen sampling. Here, we use the proportion of DNA (%) as a semi-quantitative measure of DNA amount; allowing us to distinguish the relative proportion of different plant taxa used by the honey bees.

Statistical analysis was conducted using IBM SPSS Statistics v22 and R 3.1.2. Spearman’s Rank Correlations with Bonferonni correction for multiple testing were used to assess whether each colony of honey bees used the same plants in similar proportions. Spearman’s Rank Correlations were also used to compare the amount of plant DNA within the honey to the area of occurrence of that plant species in order to investigate whether plants found at higher levels in the honey were also the most widespread within the study site. The richness and diversity (Simpson’s) of taxa recorded within April and May were compared using Mann-Whitney U tests. Spearman’s Rank Correlations and Mann-Whitney U tests were used as the data did not meet the assumption of normality required for parametric tests. The proportions of native, near native, and horticultural plants within the major plants (occurring in the honey at greater than 1%) compared to the minor plants (occurring at less than 1%) were assessed using Fisher’s exact test.

## Additional Information

**How to cite this article:** de Vere, N. *et al*. Using DNA metabarcoding to investigate honey bee foraging reveals limited flower use despite high floral availability. *Sci. Rep.* **7**, 42838; doi: 10.1038/srep42838 (2017).

**Publisher's note:** Springer Nature remains neutral with regard to jurisdictional claims in published maps and institutional affiliations.

## Supplementary Material

Supplementary Dataset 1

Supplementary Dataset 2

## Figures and Tables

**Table 1:** Colony sizes characterized by the number of frames of adult bees and brood during April and May for three hives located within the National Botanic Garden of Wales.

	Number of frames	Hive A	Hive B	Hive C
April	Bees	14	8	10
April	Brood	11	5	8
May	Bees	21	16	17
May	Brood	15	8	13

**Table 2:** Plant use compared to availability within the study site.

	April	May	April and May combined	
Number of plant families in flower		80	85	96
Number of families recorded in honey	All hives	18 (23%)	29 (34%)	31 (32%)
	Hive A	15 (19%)	14 (16%)	20 (20%)
	Hive B	11 (14%)	25 (29%)	26 (27%)
	Hive C	10 (13%)	20 (24%)	21 (22%)
Number of plant genera in flower		291	360	437
Number of genera recorded in honey	All hives	31 (11%)	45 (13%)	47 (11%)
	Hive A	26 (9%)	19 (5%)	31 (7%)
	Hive B	19 (7%)	39 (11%)	40 (9%)
	Hive C	16 (5%)	34 (9%)	35 (8%)

Supplementary Data SI 2 contains a list of all plants in flower within the study site.
